# A Tale of Two Cities in Fluorescent Sensing of Carbon Monoxide: Probes That Detect CO and Those That Detect Only Chemically Reactive CO Donors (CORMs), but Not CO

**DOI:** 10.1021/acs.joc.4c02301

**Published:** 2024-11-14

**Authors:** Dongning Liu, Xiaoxiao Yang, Binghe Wang

**Affiliations:** Department of Chemistry and Center for Diagnostics and Therapeutics, Georgia State University, Atlanta, Georgia 30303, United States

## Abstract

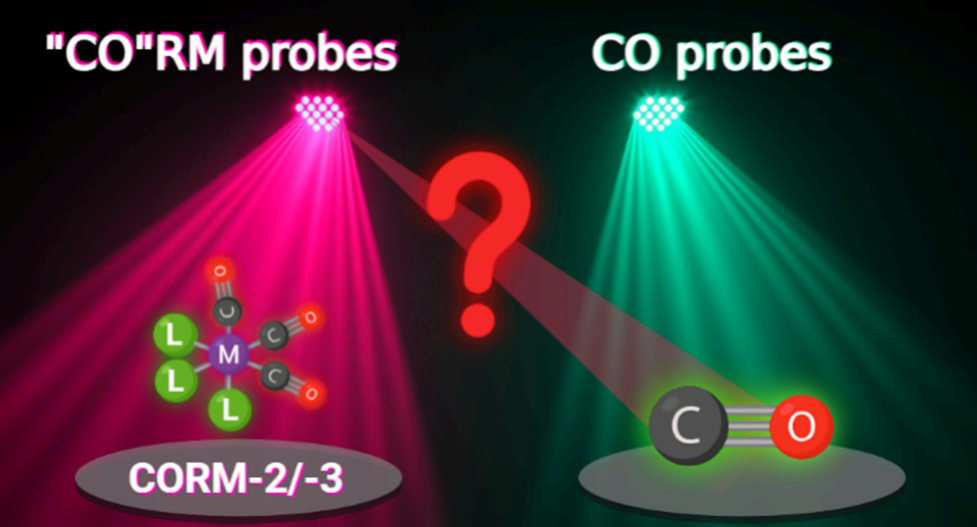

Carbon monoxide (CO) is endogenously produced with a range of pharmacological activities. Sensitive and selective detection of CO is critical to studying its biology. Since the first report of a CO fluorescent probe in 2012, more than 100 papers on this topic have appeared. Noteworthy in such work is the widespread use of two commercially available ruthenium–carbonyl complexes (CORM-2 and CORM-3) as CO surrogates. Unfortunately, these two CORMs are chemically very reactive and preferentially release CO_2_ but not CO, unless in the presence of a nucleophile. As a result, there are “two tales” of the reported CO probes: those that detect CO and those that detect only the CORM used but not CO. In addition, because of their lack of reliable CO production and fast degradation in an aqueous solution, there is the question of what “detecting CORM-2 or CORM-3” really means in the context of CO research. Additionally, for applying fluorescent CO probes in detecting low levels (often nanomolar) of CO *in vivo*, fast reaction kinetics is a prerequisite for meaningful results. In this Perspective, we discuss in detail these issues with the understanding of the evolutionary nature of scientific discoveries and the aim of preventing further confusion.

## Introduction

Recent years have seen rapidly growing interest in studying carbon monoxide (CO) as an endogenous signaling molecule and a potential therapeutic agent.^1,2^ Along this line, there is a large body of literature demonstrating (1) the endogenous nature of CO production through heme degradation by heme oxygenase (HMOX),^1−4^ (2) the large safety margins of CO in the context of drug discovery,^3^ and (3) the pharmacological effects of CO in inflammation of various types,^4^ sickle cell disease,^5^ cancer,^6,7^ cancer metastasis,^8^ cyto-protection and organ protection,^9−13^ neuromodulation and cognition via a possible CO–dopamine–heme oxygenase signaling axis,^14^ and regulation of the circadian clock.^15^ In addition, CO delivered via non-inhalation methods offers significant advantages.^3,16,17^ Therefore, there have been extensive efforts to develop various delivery methods, including metal–carbonyl complexes as CO-releasing molecules (CORMs) capable of nucleophile-, photo-, enzyme-, or redox-triggered release;^18−22^ CO foam formulations;^23^ ultrasound-sensitive CO-carrying micelles;^24^ an aqueous CO solution;^5^ and organic molecules capable of releasing CO upon photolysis (PhotoCORMs),^25−29^ X-ray irradiation,^30,31^ redox activation,^32−34^ chemoexcitation,^35^ esterase catalysis,^36,37^ or enrichment^38^ as well as immobilization or encapsulation of CO donors in a solid-phase matrix.^39,40^ Along this line, Larsen’s lab^41,42^ and our group^26,32,33,36−39,43−52^ have been working on developing organic CO prodrugs that take advantage of cheletropic CO extrusion from norbornadienone as a way to donate CO. Recently, we have also developed CO prodrugs using oxalic acid derivatives^53^ and phenylpyruvic acid (PPA) as the CO donor through decarbonylation chemistry.^54^

With all of the success, naturally there are also unique challenges in studying CO, including the need for an in-depth understanding of its molecular mechanism(s) of action,^55,56^ the difficulty in precise dosing of this gaseous molecule and in studying its dose–response relationships,^57,58^ the lack of understanding of how heme supplies meet the need for rapid signaling (though there is more than rapid signaling in CO’s actions),^59^ the “bipolar” scenario of CO’s large safety margin and the public perception of it being a potent toxin,^3^ and difficulty in studying the CO concentration in cell culture and in animal models with temporal and spatial control. The last point spurred much interest in developing CO fluorescent probes, beginning with the seminal work by the laboratories of Chang^60^ and He.^61^ Subsequently, there have been a large number of publications on CO probes of various features for different types of applications.^60−64^ Such probes have played very important roles in studying CO biology.^65,66^ There have been recent reviews summarizing this field analyzing sensitivity, selectivity, and different fluorophores used.^65,67−76^ However, there is one special aspect that deserves significant attention for the health of the CO fluorescent probe field: the literature on the use of some commercially available CO donors as CO surrogates. These CO donors are four carbonyl complexes with a transition metal ion or borane and are named “CO-releasing molecules” or CORMs: CORM-2, CORM-3, CORM-A1, and CORM-401.^44^ These CORMs have extensive chemical reactivity, which overwhelms the reactivity of CO and thus plays a dominant role in their reaction with some “CO probes”. In addition, CORM-2 and CORM-3 predominantly release CO_2_ instead of CO unless in the presence of a nucleophile. Therefore, using such CORMs has led to two types of “fluorescent probes for CO”: **those that truly detect CO and those that respond to only the CORM used but not CO**. In addition, there is no known chemistry that would suggest the ability for the latter type of probes to detect CO. In essence, this “Tale of Two Cities” emphasizes the need for fluorescent probe design to be soundly grounded in fundamental organic chemistry and chemical reactivity in general as well as the need to use a clean CO source for assessing the probes, especially if the proposed detection mechanism relies on unprecedented chemistry. In this Perspective, we discuss the various chemical reactions used for developing “CO probes” and related issues with the CORMs used, which have had a profound impact on the CO field and have significantly convoluted the data in the literature on fluorescence CO probes and CO as a potential therapeutic agent.^44^

## CORMs: The Origin of the Two Tales

**Figure 1:**
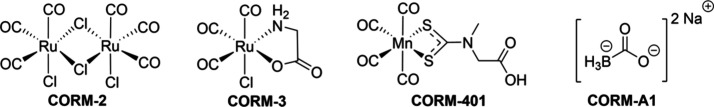
Structures of CORM-2, CORM-3, CORM-401, and CORM-A1.

The discussion of the complicated landscape in the development of fluorescent CO probes cannot begin without telling the story of the four most widely used CO donors, which are carbonyl complexes with ruthenium(II), manganese(I), or borane. These compounds were cleverly named as “CO-releasing molecules” or CORMs, with CORM-2,^77^ CORM-3,^78^ CORM-401,^79^ and CORM-A1^80^ (Figure 1) being widely available through commercial vendors. On one hand, the availability of these CO donors in the early days of the field spurred much interest in studying CO biology, presumably because the solid nature of these CORMs allows researchers to overcome the difficulties in using gaseous CO. A Pubmed search in October 2024 led to ∼690 publications that touched upon at least one of these CORMs, indicating the broad impact of these molecules. On the other hand, convolution quickly developed because of the CO-independent biological activity of these chemically very reactive carbonyl complexes. The popularity of these four CORMs as “CO surrogates” in studying CO biology has led to a large number of publications showing CO-independent biological activity from these CORMs.^45,46,48,49,51,81−89^ There is a comprehensive review on this subject.^44^ Therefore, the problems of using these CORMs for biological studies are not discussed here. Instead, this study focuses on the lack of or unpredictable CO-donating ability and the extensive chemical reactivity of these four CORMs, which are the primary reasons leading to the “Tale of Two Cities” with some probes capable of detecting CO and others capable of detecting only the CORM used as a “CO source” but not the CO itself. We make this specific attribution of problems to these CORMs because it is not reasonable or at least not practical to expect researchers to fully assess the validity of each commercially available reagent in their research, especially considering the expensive nature of these CORMs and the lack of readily available methods for end users to conduct quality assurance work.^44,50,90^ For example, the stated method for quality assessment for CORM-A1 is nuclear magnetic resonance (NMR), yet there is no characteristic NMR signature for assessing the validity and purity of CORM-A1.^50^ Similarly, CORM-401 is supposed to be a Mn(I) complex, yet it contains Mn(II) species from a commercial vendor, which can be detected only using an electron paramagnetic resonance machine, available only in specialized laboratories. In addition, the witty use of a “CO-releasing molecule” or CORM in naming each compound made it much easier for application scientists, peer reviewers, and editors to accept its face value as a CO surrogate. Recent years have seen a large number of publications clarifying many issues with these CORMs, including (1) idiosyncratic or a lack of CO production from these CORMs under nearly physiological conditions, (2) extensive chemical reactivity with organic functional groups, (3) a lack of stability and ways for end users to conduct quality assessments, (4) their susceptibility to perturbations of CO release yields and kinetics under various conditions, and (5) a lack of adequate negative controls for the non-CO-donating functions. Some of these aspects are addressed in a recent comprehensive review.^44,90^ These are all issues that impact their use in the assessment of the use of CO probes. Below we discuss these issues in detail.

### The Extensive Chemical Reactivity Constitutes a Major Problem When These “CORMs” Are Used as a “CO Donor” in Developing CO Probes

**Scheme 1:**
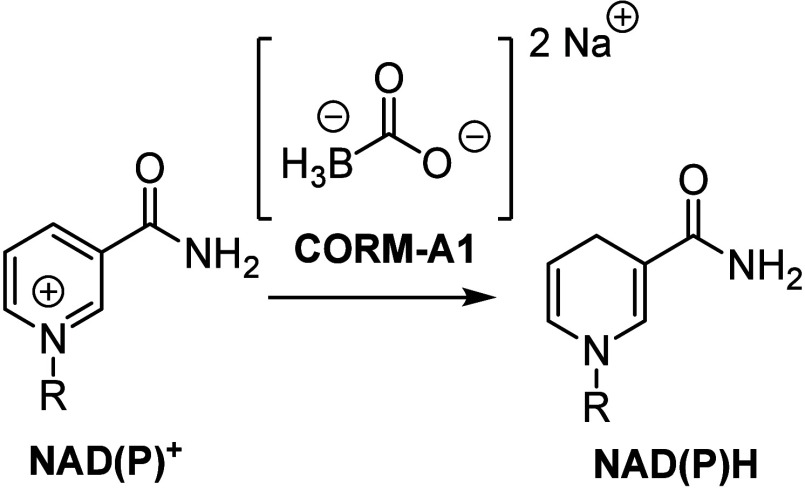
Reduction of NAD(P)^+^ by CORM-A1

**Scheme 2:**
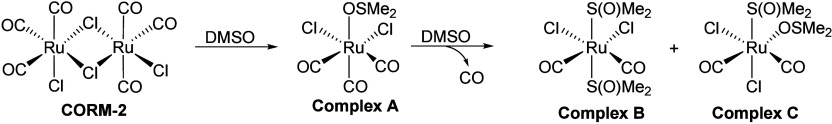
Proposed Mechanism for the Release of CO from CORM-2 in DMSO Adapted from ref (99). Copyright 2017 Elsevier.^16,47,99^

**Scheme 3:**
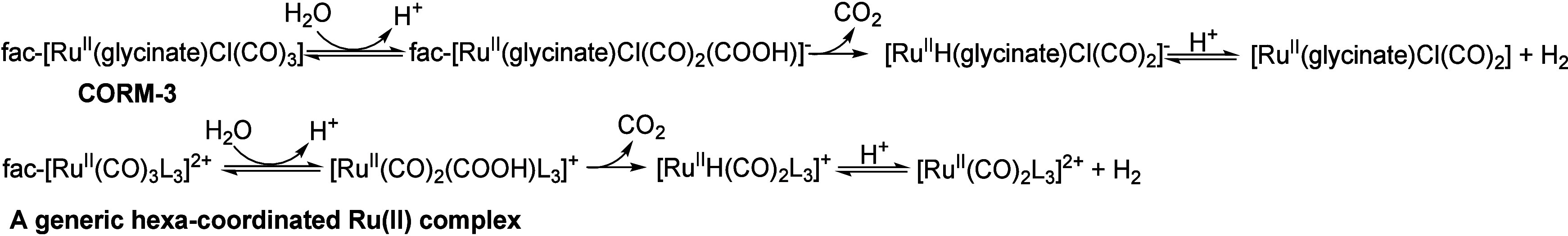
Proposed Mechanism for the Release of CO_2_ (water–gas shift reaction) from CORM-3 (top) and Originally Proposed Generic Mechanism with L Presumed to be Charge-Neutral (bottom) Partially adapted from ref (16). Copyright 2015 The Royal Society of Chemistry.^16,99,100^

A quick glance at the structures of these four CORMs would suggest chemical reactivity issues (Figure 1). For example, CORM-A1 is a carbonyl complex of borane, a textbook case of a strong reducing agent. Borane complexes are known to readily reduce carboxylic acids, ketones, amides, nitriles, aldehydes, alkenes, and alkynes, among many other compounds (Table 1).^91^ In addition, CORM-A1 has also been reported to reduce NAD(P)^+^ (Scheme 1).^50^ For ruthenium-based CORM-2 and CORM-3, the level of complexity is even higher. Ruthenium complexes are known to be very reactive.^16,51,92,93^ In the case of CORM-2 and CORM-3, they have been reported to reduce functional groups such as the nitro group and nitrite and catalytically degrade hydrogen peroxide (as a catalase mimic) (Table 2).^94−96^ Specifically, CORM-2 and CORM-3 have been studied using three different substrates to show their ability to reduce an aromatic nitro group under nearly physiological conditions.^94^ In addition to their ability to function as reducing agents, ruthenium complexes have been found have catalase-like activity in catalyzing the degradation of H_2_O_2_. Such a reaction is very fast, allowing for almost complete degradation of 5 mM H_2_O_2_ within 10 min in the presence of 20 μM CORM-2.^95^ Given the proposed redox signaling role for CO, the redox chemistry of CORM-2 and CORM-3 is expected to be problematic beyond the chemistry issues. In addition, CORM-2 and CORM-3 have been shown to undergo decomposition in DMSO (Scheme 2) and the water–gas shift reaction to produce CO_2_ instead of CO in water, saline, various buffers, and cell culture media (Scheme 3).^16,44,52,97,98^ This point is discussed in detail in the section on CO production.

**Table 1:** Products of the Reduction by Borane Complexes

reagent/substrate	imine	acyl halide	aldehyde	ester	amide	carboxylic acid
NaBH_4_	amine	–a	alcohol	–a	–a	–a
NaCNBH_3_	amine	–a	alcohol	–a	–a	–a
Na(AcO)_3_BH	amine	–a	alcohol	alcohol	amine	–a
B_2_H_6_	–a	–a	alcohol	alcohol	amine	alcohol
Li(Et)_3_BH	–a	alcohol	alcohol	alcohol	alcohol	–a

a No reaction or no productive reaction.

**Table 2:**
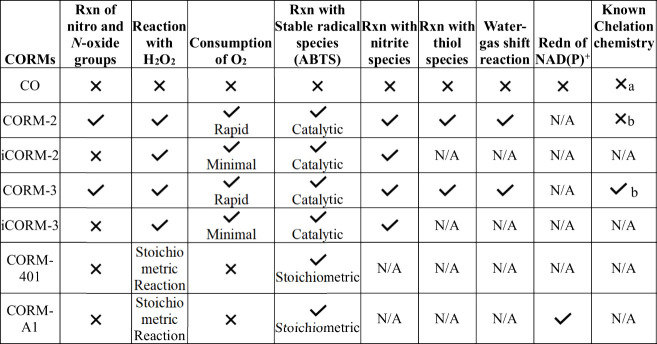
Summary of the Chemical Reactivities of the Four Commonly Used CORMs Compared to Those of CO under Nearly Physiological Conditions

a Chelates certain transition metals.

b Chelates with organic functional groups such as thiol species.

CORM-401 is a carbonyl–Mn(I) complex (Figure 1). It is well-known that Mn(II), Mn(IV), and Mn(VII) are the most common stable oxidation states.^101,102^ Therefore, CORM-401 is in an unstable redox state and is known to be a reducing agent capable of scavenging free radicals, reducing H_2_O_2_, reacting with thiols, and spontaneously degrading to Mn(II) species among others.^90^ Specifically, CORM-401 was found to react with H_2_O_2_ and ABTS [2,2′-azino-bis(3-ethylbenzothiazoline-6-sulfonic acid)] in a noncatalytic fashion.^95^

Table 2 lists the known chemical reactivities for these four CORMs. These reactions generally fall into four categories: redox reactions, including reaction with organic functional groups and molecular oxygen; chelation/reaction with a nucleophile, including proteins; catalytic degradation, including peroxide; and self-degradation/stability issues in an aqueous solution, including reactions such as the water–gas shift reaction and oxidation. Though there are overlapping reactivities among these four categories, they represent different types of problems for which to watch. With such extensive chemical reactivity, it is easy to recognize the extreme complexity when such a CORM is used as a “CO surrogate” for assessing a CO probe, even if they could produce CO in a predictable fashion. (They do not. See below.)

### The Lack of Predictable CO-Donating Ability Is Another Problem with These CORMs

Though these compounds are named as CO-release molecules or CORMs, they do not release CO under nearly physiological conditions, release only a miniscule amount of CO, or release CO in an unpredictable fashion.^44,50,90^ Coupled with their strong and extensive chemical reactivity, these are characteristics that do not conform to the name “CO-release molecules”. Indeed, Poole and co-workers have suggested that CORM-2 is a misnomer.^47^ We agree with such an assessment. CORM-3 is the same.^46,48,49,81,103^ Furthermore, CORM-A1 and CORM-401 have many of the same traits unbefitting to be called a “CO-releasing molecule” in the context of studying CO biology.^44^

Specifically, CORM-2 and CORM-3 are the two most commonly used CO donors in studying CO probes, produce no or a miniscule amount of CO unless in the presence of a nucleophile, and have extensive chemical reactivity. This represents a key issue in generating convoluted results in the CO probe field. When CORM-2 and CORM-3 were introduced as “CO-releasing molecules” ∼20 years ago, there seemed to be rigorous work in determining the level of CO production using a myoglobin assay based on its spectroscopic changes after CO binding. Sodium dithionite was used in determining the CO production yield. Later, dithionite turned out to be the key culprit, leading to much confusion in the field. Specifically, sodium dithionite was used as a reducing agent to keep the myoglobin molecule in the reduced ferrous state for CO binding. However, it did more than keeping myoglobin in the reduced state; it also triggered the release of CO from CORM-2 and CORM-3. Many later studies revealed the lack of CO production without dithionite or another nucleophile such as a thiol or DMSO.^16,22,46,47,51,98,104^ We also conducted validation experiments with similar findings.^97^ We further demonstrated (CORM-2/-3 – iCORM-2/-3) ≠ CO, where iCORM-2 and -3 represent the commonly used “negative control” derived from “depleting” CO from the corresponding CORM. Incidentally, the same dithionite issue plagues the study of two other commercially available CORMs, CORM-401^90^ and CORM-A1,^50^ which possess similar issues of unpredictable CO-release properties and extensive chemical reactivity. In addition, Romão and colleagues unequivocally demonstrated that Ru-based CORM-2 and CORM-3 produced no CO in H_2_O, saline, PBS (pH 7.4), or RPMI medium with or without FBS; instead, they undergo a water–gas shift reaction (WGSR), producing CO_2_ instead of CO with the concomitant formation of molecular hydrogen (Scheme 3).^16,98^ As such, there is no real meaning in detecting CORM-2 and CORM-3 in the context of a CO-related search. Importantly, these CORMs degrade very fast, leading to a complex mixture and further complicating the concept of “detecting CORM-2/-3”. It should be noted that the WGSR has been known for a long time. First reported by Felice Fontana in 1780, the WGSR is a very important industrial process for the production of molecular hydrogen.^105^ Specific to the subject of this Perspective, the WGSR involving homogeneous Ru catalysis was first reported in the 1970s and has been extensively studied by Peter Ford.^100^Scheme 3 (top section) shows a proposed mechanism for the WGSR involving CORM-3 based on the work of Poole,^47^ Romão,^16^ and Ford.^100^ The bottom portion of Scheme 3 shows a generic mechanism for the WGSR involving similar Ru complexes as proposed by Romão.^16^ In such a mechanism, the catalytic oxidation of CO to CO_2_ is accompanied by the formation of a formal hydride species, which reacts with a proton to produce molecular hydrogen. It is ironic that the name “CO-releasing molecule” or “CORM” continues with molecules that do not reliably release CO. In addition, the continuing marketing of such “CO-releasing molecules” as CO surrogates by major vendors perpetrates the notion of such molecules being “CO equivalents” or “CO surrogates” for those who lack the tools or knowledge needed to assess their chemical properties.

Table 3 summarizes the “CO-release” properties (or lack thereof) of these four CORMs. These traits in the absence of or unpredictable CO production coupled with their extensive chemical reactivity dictate the need to use alternative sources of CO for fluorescent probe development and the necessity to include CO gas as a positive control in validation work, with special attention to the magnitude of response and reaction kinetics.

**Table 3:** “CO-Release” Properties (or lack thereof) of These Four CORMs

CORM	instrument/method for CO detection	summary of results	ref
CORM-2	myoglobin assay (with dithionite)	0.7 mol of CO detected (measured as COMb) per mole of CORM-2	(106)
	electrode, myoglobin assay (with dithionite)	no CO detected using CO an electrode, only with the myoglobin assay in the presence of dithionite	(107)
	GC-TCD and gas-phase IR	only CO_2_ detected from CORM-2, not CO	(16)
CORM-3	myoglobin assay (with dithionite)	1 mol equiv of CO detected (as COMb) (*t*_1/2_ = 4–18 min) at pH 7.4 under nearly physiological conditions	(78)
	GC-TCD	no CO production detected, only CO_2_ (except in H_2_O at pH 1.9 where neither was detected)	(52)
	GC-TCD	1 equiv of CO_2_ detected after 24 h from CORM-3, not CO	(98)
CORM-A1	myoglobin assay (with dithionite) and CO electrode	(1) 1 mol equiv of CO detected via the Mb assay	(50), (80)
		(2) low and highly variable CO yield (0.5–15%) in the first 15 min unless in the presence of an added reagent	
		(3) *t*_1/2_ (at 37 °C) using the MB assay: 2.5 min at pH 5.5 and 21 min at pH 7.4	
		(4) *t*_1/2_ (at 37 °C) using CO electrode: 2 min at pH 5.5 and 27 min at pH 7.4	
		(5) significant pH, temperature, redox, buffer, and buffer concentration dependence observed	
	gas-phase FT-IR	(1) ≤0.91 ± 0.09 mol of CO per mole of CORM-A1	(108)
		(2) highly dependent on pH	
CORM-401	GC and myoglobin assay (with dithionite)	(1) wide variability of CO yield (0.13–1.64 equiv in 15 min) depending on conditions and the presence of other chemicals such as H_2_O_2_, albumin, dithionite, and buffer component(s)	(90), (109)
		(2) 0.33 mol equiv of CO by GC after 4 h in PBS buffer	
		(3) 3.2 equiv of CO detected after 15 min by the Mb assay	

## Reaction-Based CO Probes: How the Two Stories Have Unfolded Thus Far

**Scheme 4:**
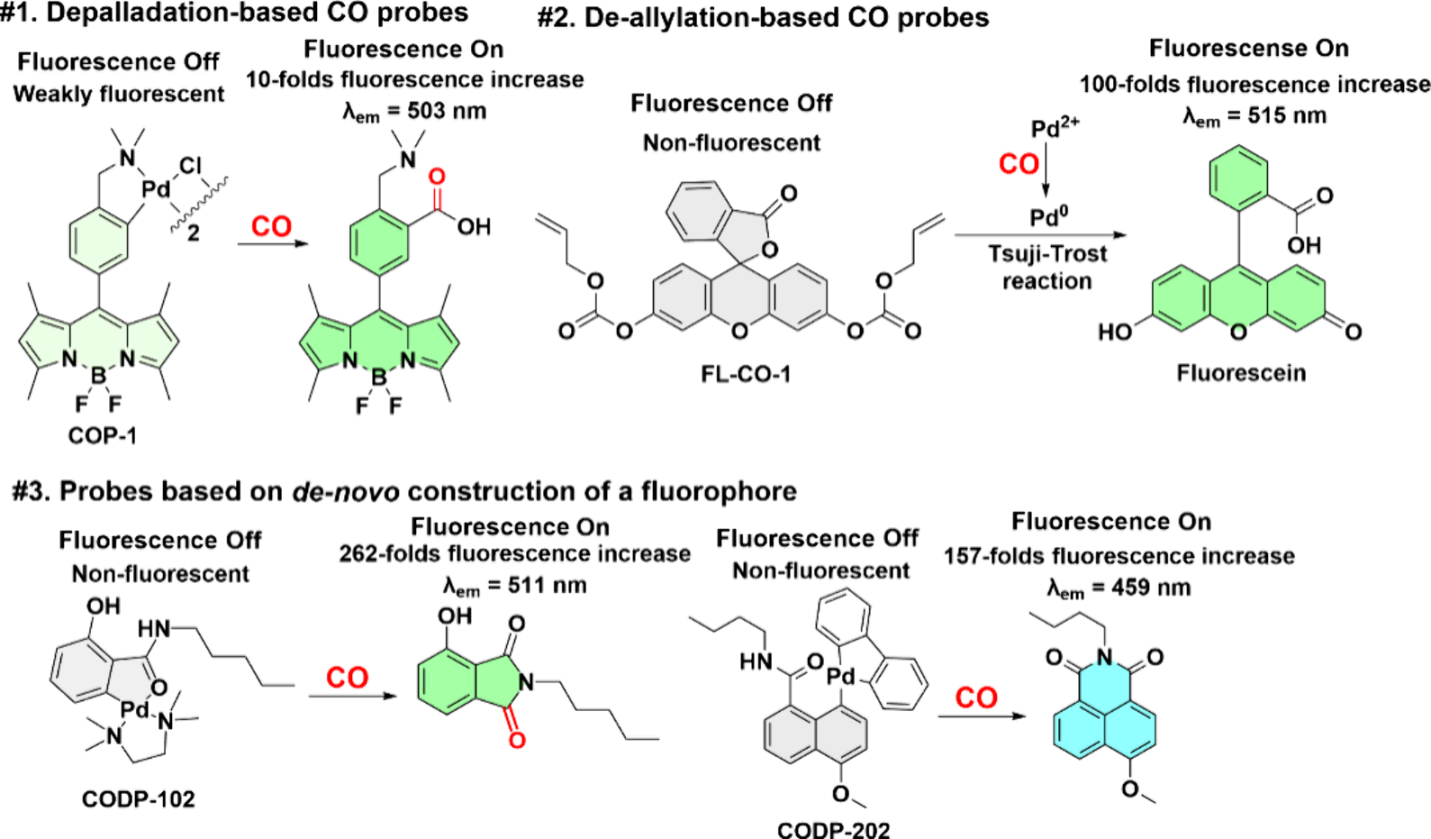
Examples for Three Types of CO Probes (#1–#3) Based on Pd-Mediated Reactions

Among all of the reaction-based probes, there are seven types of “reactions” that form the “foundation” of the chemical design principles. Scheme 4 shows the concepts and examples of palladium-mediated reactions used in the design of fluorescent CO probes.^60,110,111^ Such probes use well-precedented chemistry, including (1) unmasking a fluorophore via a palladium-based carbonylation–dequenching method (#1 in Scheme 4), (2) fluorescence turn on based on CO reduction of Pd(II) to generated Pd(0) for catalyzing deallylation and subsequent fluorescence turn on (#2 in Scheme 4), and (3) Pd-mediated *de novo* construction of a fluorophore through the same type of carbonylation (#3 in Scheme 4) as that of example #1. Table 4 summarizes key features of these probes.^60,112−119^

**Table 4:** Summary of the CO Probes Based on Known Pd-Mediated Reactions

entry	CO probes	type of CO-sensing chemistry	prior precedents of the same reaction	CO source(s) used	comments
1	COP-1, CC-CO, ACP-1 and -2, 1-Ac, HFCO-1, ANRP, Nile red CO probe, COP-3E-Py, MeNap-Pd, NAP-PMA, Nap-Pd, BOD-Pd, TPANN-Pd, BTBM, Pd-BNP-OH^60,112−125^	carbonylation-initiated depalladation and dequenching^60,112−125^	palladium-mediated carbonylation^126−128^	CORMs, CO gas	(1) widely used to show sufficient sensitivity
					(2) the side reaction with thiol can significantly impact utility
					(3) fifteen papers published using similar strategies
2	NBD-APC, FL-CO-1, Ratio-CO, MPVC-II, DCPO-probe 1, CyAPC, LW-CO, FR-CO, BTCV-CO, DCI-CO, P1, DCX-CO, two-site BODIPY probe, FDX-CO, FDX-CP, CP-CO, Mito-NIB-CO, HPQ-BI-CO, PMAH–CO, HPQ-MQ-CO, RTFP, THBTA-CO, COP, CO-H1, XPd, TBM-CO, Probe 1, QCy7-CO, PCO-1, BTHC–CO, Cou-CO,CDCI-CO, ER-NapAllyl, Mito-Ratio-CO, CyD1-UCNPs@mSiO_2_, Hcy-CO, DOP-CO, MTR-CO, CO-B, Rh-NIR-CO, NF-APC, NIR-Ratio-CO, TCF and DCI, Gol-CO, MTIPIM-CO, CORM3-AE, Allyl-luciferin, 7′-iodo-luciferin^62,63,129−176^	Pd(0)-mediated deallylation reaction to release a fluorophore^62,63,129−176^	Tsuji–Trost reaction^177,178^	CORMs, CO gas	(1) short synthetic route
					(2) using existing fluorophores
					(3) slow kinetics
					(4) the insoluble nature of Pd(0) may present “localization” problems *in vivo*
					(5) fifty papers published using similar strategies
3	CODP-102, -103, -106, -202^64^	probes based on the *de novo* construction of a fluorophore^64^	palladium-mediated carbonylation–lactamization^179,180^	CO gas, CO prodrug, CORMs	(1) zero background, fast kinetics, and exclusivity in CO detection
					(2) long synthesis
					(3) stability issues with some

**Scheme 5:**
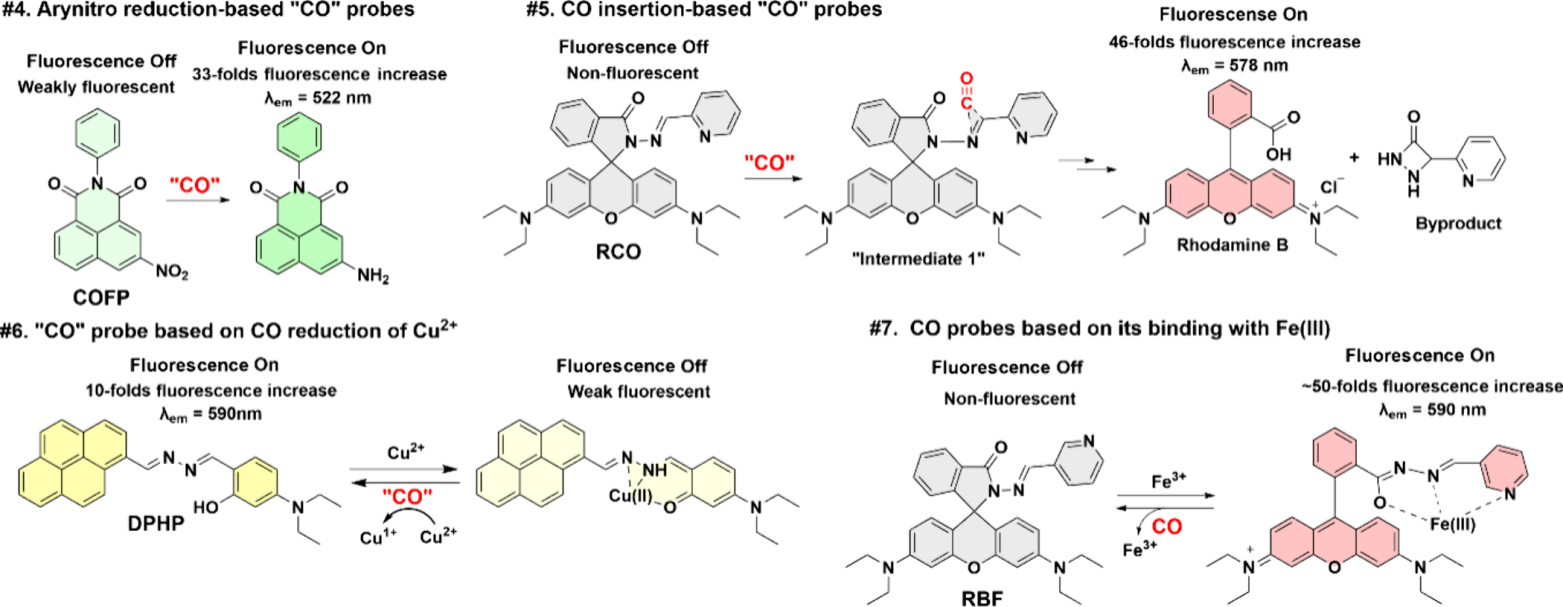
Examples for Four Types of “CO Probes” (#4–#7) Based on Unprecedented Chemistry

In addition to the palladium-based chemistry, there are also approaches that use otherwise unknown chemistry at ambient temperature, including “reduction of an arylnitro group” by CO (#4 in Scheme 5), uncatalyzed “CO insertion” into a double bond (#5 in Scheme 5), “reduction of Cu(II) to Cu(I)” by CO (#6 in Scheme 5), and “CO binding to Fe(III)” (#7 in Scheme 5). These approaches are conceptually described in Scheme 5 with representative examples summarized in Table 5.

**Table 5:** Summary of CO Probes That Were Designed on the Basis of Unprecedented Reactions Involving CO

entry	representative CO probe	proposed sensing mechanism	CO sources used
4	COFP^181^	reduction of an arylnitro group^159,181−202^	CORM-2, CORM-3
5	RCO^203^	uncatalyzed “CO insertion” into a double bond^203−206^	CORM-3, CO gas
6	DPHP^207^	CO “reduction of Cu(II)”^207−214^	CORM-3, CO gas
7	RBF^215^	binding of CO to Fe(III)^215^	CORM-3, CO gas

Below, we discuss the salient features of all seven types briefly.

In the first tale of reaction-based CO probes, we start with those that are based on palladium chemistry and are capable of detecting both gaseous CO and CO from a donor.

### Palladium as a Fluorescence Quencher (#1 in Scheme 4)

The mechanism of CO sensing by such probes can be broken down into two components: (1) the ability of Pd to quench the fluorescence of the fluorophore (e.g., BODIPY) used and (2) removal of Pd via Pd-mediated carbonylation of the fluorophore, leading to fluorescence turn on.^60^ There are slight variations of the sensing mechanism in the design of similar CO probes.^112^ Using Chang’s COP-1 as an example (#1 in Scheme 4), oxidative carbonylation led to a 10-fold increase in fluorescence intensity for COP-1 when studied in a 5% DMSO/95% PBS solution. COP-1 was also studied in HEK293T cells. Specifically, incubation with CORM-3 (5 and 50 equiv) and COP-1 (5 μM) led to increases in fluorescence intensity of 1.7- and 2.5-fold, respectively. The probe showed a good level of selectivity over other biologically relevant reactive oxygen and reactive nitrogen species, including H_2_O_2_, tBuOOH, NaOCl, O_2_•^–^, NO, and ONOO^–^. There is a comprehensive summary of available CO probes up to 2022 by Michel and co-workers, which describes in detail the traits and properties of COP-1 and other probes.^65^ At this time, it is important to discuss the source of CO, especially when quantitative assessments are involved. In this case, CORM-3 was used as the CO source and DMSO was used as an organic cosolvent, which allows for some release of CO from CORM-3. The impact of the problem identified with CORM-3 on this specific study using COP-1 is that the stated LOD is likely a significant underestimate of its true sensitivity. However, this did not affect the fundamental message; i.e., COP-1 is a very useful fluorescent CO probe. The same is probably true with many other similarly designed CO probes when CORM-3 was used as the sole CO source with an organic cosolvent present, allowing for some CO release. Along a similar line, Lin et al. developed another Pd-mediated CO probe named CC-CO in 2014.^112^ Upon reaction with CO, the Pd–C bond in CC-CO breaks, turning on the fluorescence. Unlike COP-1, this process does not yield a carbonylation product. There are 15 publications of such CO probes based on similar mechanisms as summarized in Table 4 (entry 1). It should be noted that many such CO probes, including COP-1, have been studied using CO gas and organic CO prodrugs, validating their ability to detect CO regardless of the source.^53^

### Deallylation-Based CO Probes (#2 in Scheme 4)

Palladium-mediated deallylation chemistry is well-known and has been applied in protein activation and living cells.^216,217^ Such reaction requires Pd(0) as a catalyst.^177,178^ Incidentally, CO has been reported to reduce Pd(II) to Pd(0) under ambient conditions.^218^ Therefore, there have been CO probes that take advantage of these two sequential reactions for fluorescence turn on. As such, for the deallylation-based probes to sense CO, at least 1 equiv of Pd^2+^ (PdCl_2_) is needed to react with CO, leading to the generation of Pd^0^ and then the subsequent deallylation as a trigger for fluorescence turn on. As shown in Table 4, there are 50 publications on CO probes based on this mechanism; they contain different core fluorophores such as rhodamine, NBD (nitrobenzofurazan), and BODIPY, among others. As an example, Feng’s group reported a rhodamine-based CO probe, FL-CO-1 (#2 in Scheme 4).^62^ FL-CO-1 has a reported LOD as low as 37 nM with a high signal-to-noise ratio (>100-fold) in 10 mM PBS buffer [pH 7.4, with 0.5% (v/v) DMSO]. When examined against common anions (F^–^, Cl^–^, Br^–^, I^–^, SO_4_^2–^, and AcO^–^), thiols (Cys, Hcy, and GSH), and reactive oxygen species (ROS) (ClO^–^, H_2_O_2_, ROO•, tBuOO•, and •OH), FL-CO-1 showed selectivity in the range of 40–100-fold (based on the difference in the fluorescence intensity). In an *in vitro* study, A549 cells were preincubated with different CO sources: CORM-3 (1, 5, and 10 μM), a CO gas solution (50 μM), or heme (100 μM) (as an inducer of the enzyme, heme oxygenase-1, responsible for cellular CO production) for 30 min. Then the cells were incubated with a mixture of FL-CO-1 and PdCl_2_ (premixed, 1/1 ratio, 1 μM in PBS buffer with 0.5% DMSO). A 5 μM CORM-3 solution and a 50 μM CO solution were shown to turn on the fluorescence of the probe within 30 min. FL-CO-1 was recognized for its low background, high sensitivity, and selectivity for carbon monoxide (CO). In early 2014, Zhang et al. developed an innovative dual-mode CO probe based on nitrobenzoxadiazole (NBD), which allows CO detection both colorimetrically and fluorescently and thus visual identification by the naked eye.^63^ However, several potential limitations exist, including the toxicity of Pd^2+^,^219^ the formation of Pd(0) due to the presence of other reducing agents,^220^ and stability issues when exposed to esterase within cells.^221^ It is also possible that CORM-3 can reduce Pd(II) to Pd(0) and thus affords a higher level of signal than CO at a comparable concentration. For these kinds of probes, there are around 50 publications (Table 4). In addition, deallylation tends to happen on the scale of hours.^222^ Therefore, an additional issue deserving some attention is the kinetics of a deallylation reaction and how this might affect sensitivity.

### Probes Based on *De Novo* Construction of a Fluorophore (#3 in Scheme 4)

Building upon the carbonylation chemistry described in the first CO probe publication by the Chang lab,^60^ our lab developed a new type of Pd-mediated CO probes. We used CO as a building block so that the sensing process is the *de novo* construction of a fluorophore (Scheme 4).^64^ This approach allows for exclusivity in CO detection with no false-positive detection, very high sensitivity because of the lack of background issues, and a high dynamic range represented by the wide linearity in the fluorescence response.^103,223−227^ Specifically, these CO probes showed no inherent background signal, and the maximal magnitude of fluorescence turn on is limited only by the concentration of the probe used in the assay. For three representative probes (CODP-102, -103, and -106), the sensitivity represented by the LOD was determined as 0.45–0.9 nM by using CO solutions as the CO source. In addition, the probe can also detect CO in air as low as 0.1–0.2 ppm when using the headspace method. On the contrary, the CO sensing kinetics is shown to be very fast, with a second-order rate constant of 220 M^–1^ s^–1^ for CODP-106 (#3 in Table 4). These are reaction rates on the scale of fast click reactions such as that between a strained alkyne and tetrazine.^228^ This fast kinetics is also very important for detection sensitivity for this gaseous molecule (CO).

As a brief summary of the probes based on Pd chemistry, their design principles are based on well-precedented CO-mediated reactions. Therefore, these probes can detect both CO gas and CO from a donor as one would expect. There are a total of 66 publications of CO probes based on Pd-mediated chemistry, constituting the majority of the CO probe literature.

In the second tale of reaction-based CO probes, there are those that are based on chemistry that not only is unprecedented but also lacks a commonly accepted reaction mechanism for CO detection. Specifically, fluorescent CO probes that are based on “the ability for CO to reduce an arylnitro group” (#4 in Scheme 5), uncatalyzed “CO insertion” into a double bond (#5 in Scheme 5), “reduction of Cu(II) to Cu(I)” by CO (#6 in Scheme 5), and “CO binding to Fe(III)” (#7 in Scheme 5) are reported. As discussed in detail in the next few sections, later experiments raised serious doubts about the ability of these probes to detect CO, as one would expect on the basis of the proposed chemistry principles. We feel much of the confusion can be attributed to the widely propagated belief that the commercially available CO-releasing molecules (or CORMs) donate CO reliably and are otherwise benign and unreactive. Of course, neither was proven to be true through rigorous chemical studies.^44,46,47,50,52,90,97^

Below, we describe each briefly to highlight the improbable chemistry proposed and their ability to detect only CORM but not the CO itself. Again, Scheme 5 shows conceptually the design principle of each type of “CO probe” with examples. Table 5 summarizes the literature in each category.

### Arylnitro Reduction-Based CO Probes (#4 in Scheme 5)

**Scheme 6:**
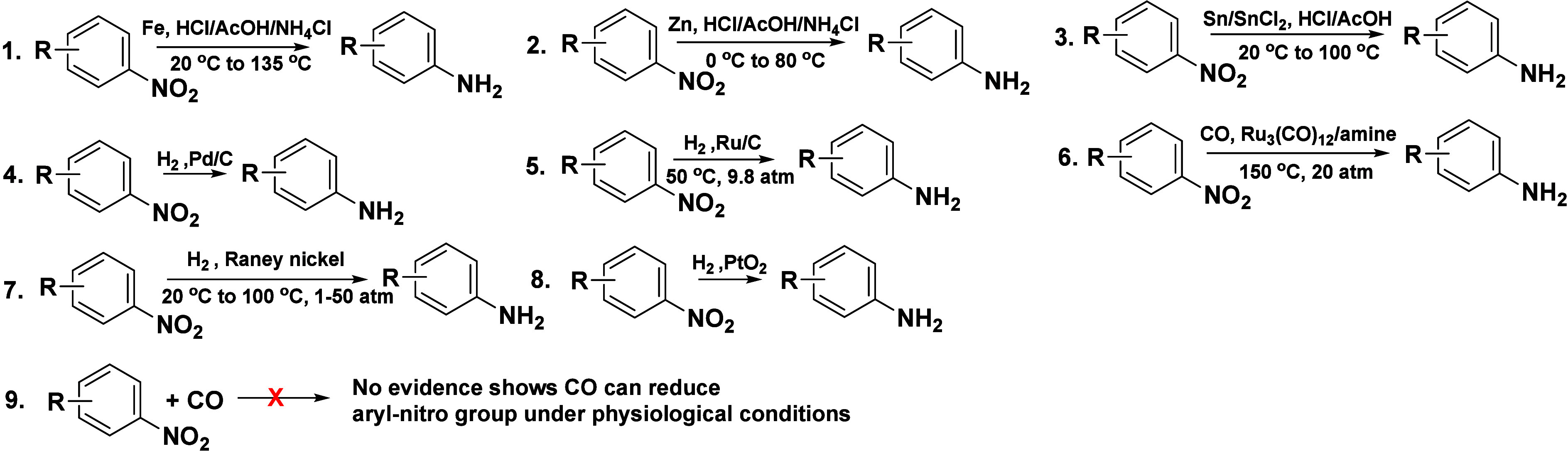
Examples of Known Reduction Reactions of Arylnitro Compounds

In 2018, it was first reported that fluorescence turn on was achieved through the reduction of an arylnitro group by “CO” under nearly physiological conditions.^181^ Chemically, the reduction of an arylnitro group is not an easy process. Known and commonly used methods for reducing an arylnitro group to an amino group usually require harsh conditions such as Fe/HCl, Zn/HCl, Sn/HCl, and H_2_/Pd.^229^ In addition, ruthenium^230^ and other metals^231,232^ have also been used as a catalyst for such reduction. Scheme 6 shows some examples and the harsh conditions required for the reduction of a nitro group. There is no known organic molecule capable of reducing a nitro group under ambient conditions. Indeed, if CO were able to reduce an arylnitro group, it would be a tremendously useful reaction and would spare the use of harsh conditions for such a transformation. Unfortunately, such reduction by CO does not happen. Since 2018,^181^ there have been 23 publications describing CO probes based on the “reduction of a nitro group by CO” (#4 in Table 5). Some of these papers specifically stated that the probes are for the detection of CORM-2 and -3, and others generally identified them as CO probes, though only CORM-2 and/or CORM-3 was used as a CO source. The proposed “CO reduction of a nitro group” has no chemistry precedent and no plausible reaction mechanism. Indeed, rigorous chemistry studies later confirmed the inability for CO to reduce a nitro group under the described experimental conditions.^94^ This was done with the “CO probe” described in the original publication and other arylnitro compounds.^94^ Specifically, exposing all of these compounds to gaseous CO at a level much higher than the CORM concentration did not cause any change or reaction, as one would expect. However, incubation with CORM-2 or CORM-3 indeed led to the reduction of the arylnitro group in a range of substrates. Studies using CORM-A1 and CORM-401 led to the same results that were seen for gaseous CO. All such results point to ruthenium chemistry from CORM-2 or CORM-3 being responsible for the observed reduction of a nitro group. Incidentally, ruthenium–carbonyl complexes have long been known to catalyze reduction of a nitro group by CO^233^ (Scheme 6, entry 6) or by hydrogen.^234^ Therefore, the conversion of a nitro group to an amino group by CORM-2 and -3 could be through either direct reduction or catalytic action by Ru(II). In both scenarios, it is the ruthenium chemistry that is the critical factor, not CO. These literature reports explain why such arylnitro-based “CO probes” were able to detect CORM-2 and -3, but not CO gas or CO from other donors such as CORM-A1 or CORM-401.^94^

### CO Probes Based on Uncatalyzed “CO Insertion” into a Double Bond (#5 in Scheme 5)

**Scheme 7:**
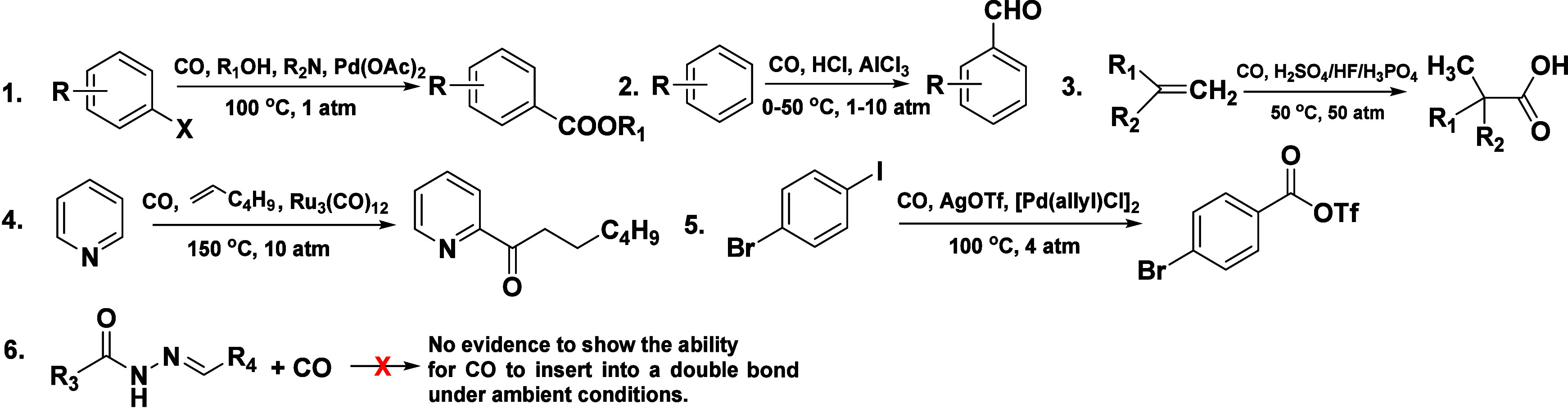
Examples of Known CO Insertion Reactions

CO insertion reactions are very useful in synthetic chemistry for the introduction of a one-carbon unit. However, because of the chemically inert nature of CO under ambient conditions, there have been extensive efforts in the search for methods for catalytic carbonylation. Transition metal-catalyzed carbonylation (CO insertion), pioneered by Heck and co-workers (Scheme 7, entry 1),^235^ has been a widely studied topic for years, enabling access to a wide range of carbonyl-containing compounds (Scheme 7).^236^ The challenge of finding an appropriate catalyst for various types of CO insertions is not a trivial issue. In industrial processes, carbonylation uses a strong acid coupled with high CO pressure to achieve carbonylation [the Koch process (Scheme 7, entry 3)].^237,238^ In 2019, a CO probe (RCO) based on uncatalyzed “CO insertion” into a hydrazone double bond was reported.^203^ In the originally proposed mechanism, “CO” simply inserts into a hydrazone double bond, leading to a few steps of peculiar rearrangements to release a fluorescent product and a byproduct with a strained four-membered ring. Interestingly, little structural evidence was provided for such an unprecedented reaction. Later studies using RCO and eight other hydrazone analogues demonstrate that the uncatalyzed “CO insertion” does not happen, as one would expect.^239^ Again, there is a large body of literature on carbonylation using CO,^236,237,240−242^ yet there is none on uncatalyzed CO insertion into any bond in an organic molecule at ambient temperature or under more forcing conditions. There are four publications of similar probes as summarized in Table 5. All of the evidence points to the inability of such probes to detect CO.

### CO Probes Based on Reduction of Cu(II) to Cu(I) (#6 in Scheme 5)

**Scheme 8:**
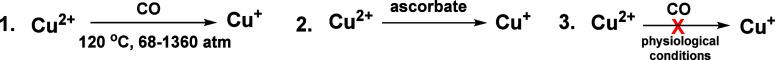
Examples of Conditions Needed to Reduce Cu^2+^ to Cu^+^

In 2022, DPHP was published as a CO probe. For its sensing mechanism, Cu(II) was said to quench the fluorescence of DPHP.^207^ “CO” was proposed to turn on the fluorescence through its reduction of Cu(II) to Cu(I) under physiological conditions (#6 in Scheme 5). It should be noted that Cu(I) is an unstable state, and reduction of Cu(II) to Cu(I) by CO had not been reported at ambient temperature. There are reports of CO reduction of Cu(II) to Cu(I), but only under forcing conditions at a higher temperature (Scheme 8).^243,244^ One would not expect the ability of CO to reduce Cu(II) under nearly physiological conditions. Indeed, extensive validation experiments with the DPHP–Cu(II) system using CO gas did not show a meaningful response to CO.^245^ On the contrary, sodium ascorbate is widely used to keep copper in the Cu(I) oxidation state; this method is widely used in copper-assisted azido–alkyne cycloaddition (click) reactions.^246^ Therefore, it was not surprising to find that the DPHP–Cu(II) system responded to ascorbate by turning on the fluorescence.^245^ Thiol was also shown to accomplish fluorescence turn on, undermining the selectivity claim for this sensing system.^245^ It should be noted that CORM-3 is a strong reducing agent.^94,96^ It is conceivable that reduction of Cu(II) by CORM-3 contributes to the fluorescence turn on of the DPHP–Cu(II) system. In any case, this system responds to only CORM-3 and other reducing agents but not to CO gas. There are in total eight publications based on this strategy.^207−214^

### CO Probes Based on CO Binding with the Fe(III) Complex (#7 in Scheme 5)

**Scheme 9:**
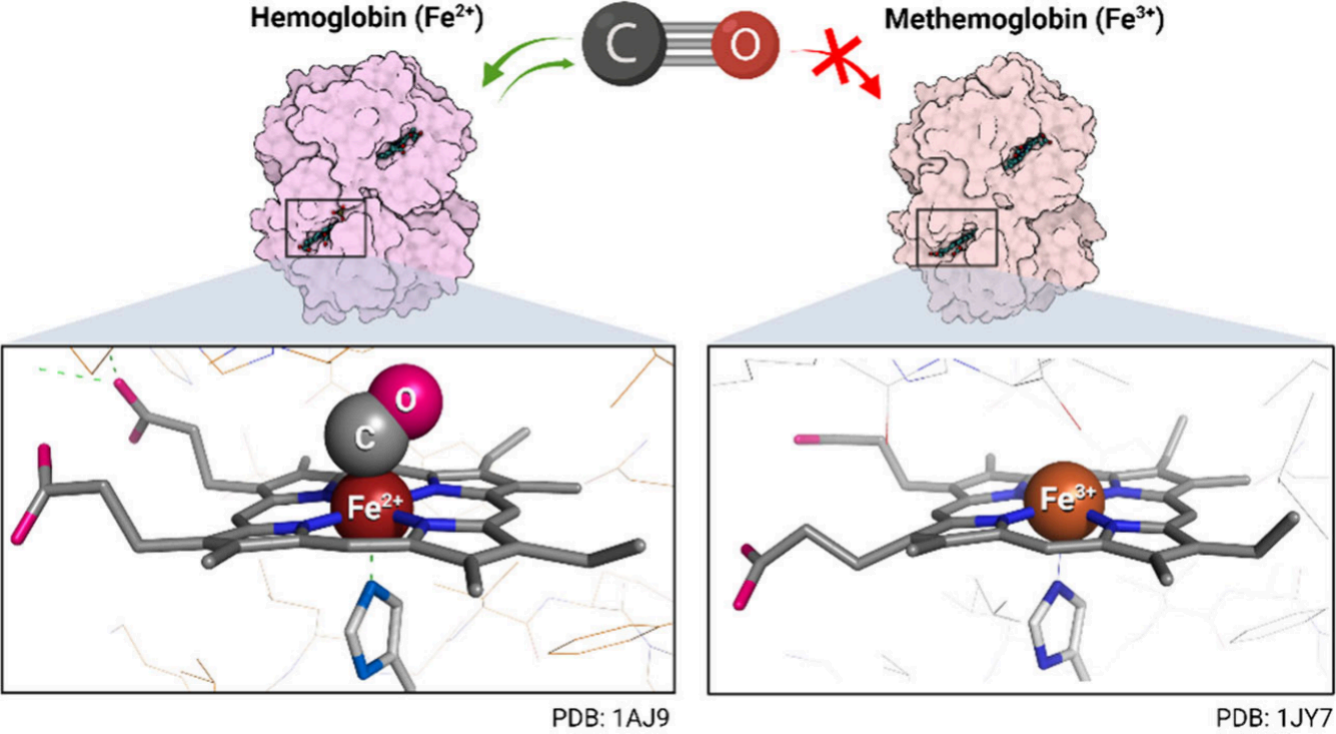
Structures of COHb in Which CO Binds to Fe(II) Protein structure rendered from Protein Data Bank entries 1AJ9 and 1JY7.

In 2024, an imine derivative of rhodamine B, RBF, was reported as a turn-off CO probe.^215^ The reaction mechanism was proposed to be through the dissociation of Fe(III) from a fluorescent complex with RBF triggered by CO (#7 in Scheme 5). The presented experimental results seem to be very sound. Again, the rigor of the experiments relied on the use of CORM-3 with verification using CO gas. However, the study did not elaborate on the mechanism of how CO (in this case CORM-3) led to the dissociation of the iron from the RBF–Fe(III) complex. The authors stated that such a design was inspired by the binding ability of heme iron with CO. However, for heme iron to bind with CO, the ferrous oxidation state of Fe is required as exemplified in the formation of carbonylhemoglobin (COHb) (Scheme 9).^247,248^ The ferric form of hemoglobin actually is not known to have the ability to bind CO.^249^ This is due to the electron configuration of Fe(II) that allows for the back bonding of the *d* orbital electron to CO, while such back bonding does not exist in the case of Fe(III). Given the inability of Fe(III) to bind with CO, it is interesting to see that such an Fe(III)-based probe was shown to respond to CO gas in the study. In addition, the linearity of the reported binding curve does not conform to what is normally expected in a binding process. In one possibility, one could envision a water–gas shift reaction involving Fe(III) and CO to give Fe(II),^250^ leading to the proposed dissociation of Fe(III) and fluorescence turn off. Though such reactions involving Fe(III) normally take place at a higher temperature, the appropriate coordination chemistry could perhaps change this. Much more rigorous chemistry studies are needed to investigate the chemical mechanism of how CO interacts with such an Fe(III) complex, if it happens as proposed. However, because there is no experimental work to examine this issue, we leave this as an open question.

As a brief summary, the use of chemically reactive CORM-2 and CORM-3 has led to many probes that respond only to the CORM used, not to CO, and the unfortunate proposal of multiple sensing mechanisms based on improbable, if not impossible, chemistry under nearly physiological conditions.

## Considerations of CO Sensing Kinetics

There is another very critical issue in CO detection using a reaction-based probe, especially in biologically relevant studies: reaction kinetics for CO sensing and/or detection. Even in simple solution-phase studies, the volatile nature of CO and its low solubility mean that the concentration changes on the scale of minutes. Therefore, for probes with reaction half-lives on the scale of hours, slow kinetics present an experimental problem for quantitative determinations. It should be noted that some of the probes require three components, while others require two components. In any case, the rate-determining step is probably a bimolecular reaction. The concentration dependency of bimolecular reactions means that the reaction rate changes with time. As such, one needs to be very careful in determining the concentration, which needs calibration curves and careful control of the experimental conditions, even without considering the volatility of CO. For cell culture and animal model studies, there are additional considerations.

Indeed, there have been many bioimaging studies of CO aimed at detecting endogenous CO generation using fluorescent probes. Such studies include detection of basal level CO, exogenous CO concentration, and after stimulation of the overexpression of heme oxygenase-1 (HO-1), which is responsible for endogenous CO production through heme degradation. For this, one can easily analyze whether there is a reasonable chance for success. The average “free” CO concentration of human tissue is estimated to be ∼2 nM [equivalent to a CO partial pressure (*P*_CO_) of 0.0019 mmHg].^59^ In mouse tissue, the endogenous CO concentrations in various organs are 2–10 pmol/mg.^251^ Though the free CO concentration is expected to vary widely throughout the body, these numbers are within a reasonable range, especially considering the *K*_d_ of hemoglobin for CO being 0.7–1.7 nM in the high-affinity form and approximately 1.8–4.5 μM in the low-affinity state.^55^ In addition, these affinity values may change depending on pH, metabolite concentrations, and other parameters, leading to changes in the CO concentration depending on the local environment. Therefore, for *in vivo* detection with any spatiotemporal control, fast kinetics becomes an even more important issue. Though the total intracellular CO concentration is the net sum of free CO and CO bound to hemoproteins, for binding considerations or reaction kinetics, only the free CO becomes part of the calculation. To determine such a low concentration of CO in a “snapshot” fashion that reflects the true concentration of CO, a CO probe must have fast reaction kinetics. We can use a hypothetical scenario to perform the calculation. When 10 μM probe is used as often seen in reported cases, the second-order rate constant needs to be at least 1160 M^–1^ s^–1^ to detect 2 μM CO within 5 min with >95% CO consumption by the probe. If such a rate is hard to achieve, increasing the probe concentration to 100 μM would require the probe to have a second-order rate constant of 116.0 M^–1^ s^–1^, which is achievable with some of the probes discussed in this review, but not all.^65^ As such, the required reaction kinetics is on the scale of fast click reactions for meaningful *in vivo* detection.^228^ There is another issue in using 100 μM probe to detect a few nanomolar concentration of CO. It requires the fluorescence intensity difference for the probe before and after fluorescence turn on to be >10 000-fold. All of these analyses assume an ideal scenario without other interference and/or background fluorescent, which may significantly change the signal-to-noise ratio. Considering these quantitative factors in bioimaging studies of CO will help enhance the robustness of the data and their interpretations. It should be noted that most of the reported approaches did not include detailed studies of the reaction kinetics with CO gas. To truly understand detection sensitivity in a biologically relevant fashion, we view including reaction kinetic data whenever possible as being very critical. Information about reaction kinetics and the concentration will help ensure proper experimental design and data interpretation in bioimaging work.

## Conclusions

With the increasing level of attention to CO’s pharmacological and signaling roles, there is a need for fluorescent CO probes for various biological studies. Along this line, there are two stories. On one hand, there has been tremendous progress made in developing CO probes for various applications. On the other hand, there are also “CO probes” that do not detect CO. These complicated problems largely originated from the use of metal–carbonyl complexes (also known as CORMs) as “CO surrogates”. The metal–carbonyl complexes have strong and extensive chemical reactivity that overwhelms that of CO, leading to identification of “CO probes” that rely on unprecedent chemistry to explain their “ability to detect CO”. In reality, these “CO probes” detect only the “CORM” used but not CO. These stories parallel that of CO-independent biological activities from these CORMs.^44^ Another challenge in using a Pd(0)-based approach is the possible uneven localization of the components (i.e., Pd, probe, and CO), especially considering the insoluble nature of Pd(0).^252^ An additional point is the question of detecting and imaging of CORM-2 or CORM-3. As discussed above, CORM-2 or CORM-3 does not reliably release CO, has strong chemical reactivity, and undergoes fast degradation in solution, leading to an intractable mixture of species. All of these findings complicate the true meaning of “detecting/imaging CORM-2 or CORM-3”, at least in the context of CO research.

Moving forward, we must consider a few important issues. First, it is critical that the chemical reactivity of a CO donor be thoroughly investigated before one would attribute the signal turn on by a CO donor to CO. Second, a clean source of CO needs to be used to confirm its detection with comparable sensitivity and kinetics. Third, all probe designs should be solidly grounded in well-known chemistry principles. Fourth, the burden is on the investigators to provide extensive structural evidence if entirely new chemistry is proposed. Fifth, in bioimaging studies, quantitative analysis involving reaction kinetics and anticipated CO concentration will be critical to the robustness of the data and proper interpretation of the results. Sixth, in multicomponent CO probe systems, reagent localization needs to be considered in cellular and animal model studies.

We hope this Perspective together with some existing excellent reviews will help prevent further confusion and help the healthy growth of this field. Especially worth mentioning are two excellent reviews that did more than summarizing the literature; they critically analyzed many other issues related to fluorescent CO probes.^65,70^

## Data Availability

The data underlying this study are available in the published article and its Supporting Information.

## Supporting Information Available

Additional details about the theoretical calculations in Considerations of CO Sensing Kinetics (PDF)
