# Supplementary material for: A Tale of Two Cities in Fluorescent Sensing of Carbon Monoxide: Probes That Detect CO and Those That Detect Only Chemically Reactive CO Donors (CORMs), but Not CO
Source: J Org Chem. 2024 Nov 14;89(24):17891–909. doi: 10.1021/acs.joc.4c02301 (PMC11667734; doi:10.1021/acs.joc.4c02301)
Supplement: Supplementary file 1 — jo4c02301_si_001.pdf [file jo4c02301_si_001.pdf]

Supporting Information

A Tale of Two Cities in Fluorescent Sensing of Carbon Monoxide: Probes That Detect CO and Those That Detect Only Chemically Reactive CO Donors (CORMs), but Not CO

Dongning Liu, Xiaoxiao Yang, and Binghe Wang*

Department of Chemistry and Center for Diagnostics and Therapeutics, Georgia State University, Atlanta, Georgia 30303, United States

*bwang31@gsu.edu

Table of contents

Theoretical calculations.. S2

Theoretical calculations

We can use a hypothetical scenario to do the calculation. When a probe is used at 10 μM as often seen in reported cases, the second-order rate constant needs to be at least 1160 $M^{-1}s^{-1}$ in order to detect 2 μM CO within 5 min with >95% CO consumption by the probe.

For the calculation:

1. The reaction is assumed as "Probe + CO → Products," with a defined 1:1 stoichiometry. The initial concentration is 10 μM for the probe and 2 μM for CO. After 5 mins, we then assume 95% consumption of CO by the probe.

2. Assuming a second order reaction, the following equation can be used. (A: Probe, B: CO)

$$\frac{1}{[A]_0 - [B]_0} \ln\left(\frac{[A]}{[B]} \cdot \frac{[B]_0}{[A]_0}\right) = kt$$

3. $[B]_0 = 2.0*10^{-6}$ M
   $[B] = [B]_0 * (1-0.95) = 1.0*10^{-7}$ M
   $[A]=[A]_0-0.95*[B]_0 = 8.1*10^{-6}$ M
   t= 300 s

4. $\frac{1}{1.0 * 10^{-5}(M) - 2.0 * 10^{-6}\,(M)} * \ln\left(\frac{8.1*10^{-6}}{1.0*10^{-7}} * \frac{2.0*10^{-6}}{1.0*10^{-5}}\right) = k * 300\ (s)$

$k = \ln 16.2 * \frac{1}{8.0*10^{-6}*300}$ $M^{-1}s^{-1}$

k= 1160 $M^{-1}s^{-1}$
